# Prevalence and correlates of psychological distress amongst healthcare workers during the COVID-19 pandemic: An online survey

**DOI:** 10.4102/sajpsychiatry.v27i0.1617

**Published:** 2021-07-29

**Authors:** Olamiji A. Badru, Kehinde O. Oloko, Abdulwasiu O. Hassan, Oyindamola B. Yusuf, Umar A. Abdur-Razaq, Saheed Yakub

**Affiliations:** 1 Department of Mental Health and Behavioural Medicine, Federal Medical Center, Abeokuta, Nigeria; 2 Department of Haematology, Haematology Mavens Specialist Center, Lagos, Nigeria; 3 Department of Medical Laboratory Science, Achievers University, Owo, Nigeria; 4 Department of Epidemiology and Medical Statistics, Faculty of Public Health, College of Medicine, University of Ibadan, Ibadan, Nigeria; 5 Department of Nursing Science, Faculty of Clinical sciences, College of Medicine, University of Lagos, Lagos, Nigeria; 6 Department of Surgery, Osun State University (UNIOSUN) Teaching Hospital, Osun, Nigeria

**Keywords:** COVID-19, psychological distress, healthcare workers, Nigeria, general health questionnaire-12 (GHQ-12), pandemic

## Abstract

**Background:**

Understanding the mental health needs of healthcare workers during coronavirus disease 2019 (COVID-19) pandemic with a view of mitigating its impact on occupational functioning is vital.

**Aim:**

To determine the prevalence and correlates of psychological distress amongst healthcare workers.

**Setting:**

The study was carried out in Nigeria during COVID-19 pandemic.

**Methods:**

A cross-sectional quantitative study using a convenience sample was conducted amongst healthcare workers. The survey comprised of two Google formatted self-report questionnaires, a 12-item General Health Questionnaire and a questionnaire containing socio-demographic, work related and knowledge of COVID-19 variables. Questionnaires were distributed via closed professional WhatsApp platforms. Data were analysed using descriptive statistics, chi-square test and logistic regression.

**Results:**

There were 313 respondents and prevalence of psychological distress was 47.0%. Females were almost twice as likely to have psychological distress as males (OR = 1.92, 95% CI: 1.21–3.04, *p* = 0.006). Healthcare workers who had no contact with COVID-19 positive patients had an 87% reduced risk of psychological distress (OR = 0.13, 95%CI: 0.23–0.25, *p* = 0.018) compared with those who had contact with COVID-19 positive patients. Healthcare workers with poor knowledge of COVID-19 had a 44% reduced risk of psychological distress compared with those with good knowledge (OR = 0.56, 95% CI: 0.34–0.93, *p* = 0.025).

**Conclusion:**

Findings revealed that healthcare workers in Nigeria reported psychological distress during COVID-19 pandemic. Greater risk was amongst females and those who had contact with COVID-19 positive patients whilst poor knowledge was protective.

## Introduction

The pandemic of 2019 novel coronavirus disease (COVID-19), caused by severe acute respiratory syndrome coronavirus 2 (SARS-CoV-2) was first reported in Wuhan, China, before becoming global.^1^ The index case in Nigeria was reported on 27 February 2020, thereafter, 10 162 cases were confirmed by 31 May 2020.^2,3^ Considering the novelty of COVID-19, the possibility of adverse effects on psychological well-being as a result of unpredictability of its outbreak cannot be overemphasised, especially amongst healthcare workers.^4^

The workplace is an environment that may generate demands and pressures such as excessive workload. Studies suggest that healthcare workers develop increased levels of psychological distress during outbreak of infectious diseases, especially when work conditions are not favourable.^4,5^ Thus, compromise of effective healthcare service delivery may be an aftermath of mental imbalance experienced by healthcare workers during COVID-19 pandemic. Contrary to the general population, healthcare workers can neither afford to stay safe at home nor practice physical distancing in view of their professional calling. In addition, scarcity of personal protective equipment exposes healthcare workers, especially clinical staff at increased risk of getting infected, which may lead to increased anxiety.^6^

A cross-sectional survey assessing psychological distress in the community and amongst medical students, medical staff and patients with COVID-19, in Iran with the Depression, Anxiety and Stress Scale (DASS) revealed lower scores amongst medical students compared with medical staff. Professionalism amongst medical staff perhaps served as a protective factor, which made them less prone to psychological distress.^7^

In addition, the emotional consequence of COVID-19 pandemic on healthcare workers in Singapore revealed that 14.5% participants had anxiety, 8.9% had depression, 6.6% were positive for stress and clinical concern of post-traumatic stress disorder (PTSD) was reported amongst 7.7%.^8^ Another cross-sectional on-line survey using the Insomnia Severity Index (ISI), the Symptom Check List-revised (SCL-90-R), and the Patient Health Questionnaire-4 (PHQ-4 to assess mental health variables amongst both medical and non-medical staff in China revealed increased morbidity amongst medical staff.^9^

Therefore, it is vital to determine the mental health impact of COVID-19 on healthcare workers, more so that healthcare workers have been reported to get infected in their line of duty.^10^ This study aimed to determine the prevalence of psychological distress amongst healthcare workers during the COVID-19 pandemic and to assess the associated, socio-demographic, level of knowledge and work**-**related correlates.

## Methods

A cross-sectional quantitative study, using a convenience sample, was conducted amongst healthcare workers between 01 April 2020 and 30 June 2020. The survey comprised of two Google formatted self-report questionnaires: A 12-item General Health Questionnaire (GHQ) to assess psychological distress and a questionnaire containing socio-demographic, work related and knowledge of COVID-19 variables. Questionnaires were sent through broadcast messages with a brief description of the study via series of network of healthcare workers’ WhatsApp groups in Nigeria. Questionnaires were then filled and submitted via the attached questionnaire link.

## Settings

The study was conducted in Nigeria from 01 April to 30 June 2020, during the COVID-19 pandemic.

### Study population

Our study population included healthcare workers residing in Nigeria who could be contacted via WhatsApp during the pandemic irrespective of the type of healthcare worker or type of health facility. Healthcare workers not residing in Nigeria were excluded.

### Sample size calculation

The sample size was estimated using the formula for determining the sample size for single proportions, which is as follows:

$$n=\frac{z_{\alpha }^{2}pq}{d^{2}}$$

where $z_{\alpha }^{2}$ = standard normal deviate at α set at 0.05 = 1.96

α = level of significance

*p* = proportion of the population with the variable of interest, that is, psychological distress

This was estimated as 35% from a previous study.^1^

*q* = 1-*p*

*d* = margin of error = 0.06

*n* = minimum sample size = 243

~ 316

Although the estimated sample size was 243, the sample size was increased by 30% (72.9) to accommodate for non-response.

### Instruments

#### Socio-demographic questionnaire

A structured questionnaire was composed to obtain information on socio-demographic and work-related variables and level of knowledge regarding the COVID-19 pandemic. The socio-demographic variables included, gender, age, type of religion, frequency of participation in religious activities and marital status. The work-related variables included, type of healthcare worker, years of service, coming into contact with COVID-19 positive patients (confirmed or suspected cases), recovery or death of COVID-19 positive patients, being a routine frontline healthcare worker (working at the first point of call in health facilities, for example, accident and emergency units) and provision of incentives as a frontline healthcare worker.

Variables to test the level of knowledge of healthcare workers towards the pandemic were: their understanding on availability of vaccine, whether it could be cured, how contagious the virus was; its prevention through: frequent hand washing, use of face masks, staying at home and social distancing. Each correct response was scored as 1 whilst a wrong response was scored as 0. The responses were added and the mean was calculated as 5.96 ± 1.05. Respondents with scores above the mean were regarded as those with good knowledge whilst those with scores below the mean were regarded as those with poor knowledge.

#### General Health Questionnaire-12

General Health Questionnaire-12 (GHQ-12) designed by David Goldberg in 1978 was used in this study.^11^ It is a 12-item easy to use screening instrument for general psychiatric morbidity such as psychological distress. Questions in the instrument were: ability to concentrate, loss of sleep over worry, playing useful part in things, ability to make decisions, being under strain, inability to overcome difficulties, ability to enjoy day to day activities, being able to face up to problems, feeling unhappy or depressed, losing self-confidence, thinking of self as worthless and feeling reasonably happy, all things considered. The instrument can be scored either on the Likert scale or on the bimodal scale. The bimodal scale was used in this study.

On the bimodal scale, the two least symptomatic answers score 0 and the two most symptomatic answers score 1 (i.e. 0-0–1-1), the score range is 0–12 with a score of 3 or more indicative of psychological distress.

The GHQ-12 has been reported to have inter-rater reliability and internal consistency with Cronbach’s alpha of 0.76.^12^ It has equally been validated for use in Nigeria^13^ and has been used in several studies to identify psychiatric distress in other settings.^14,15,16^

### Procedure

Google formatted forms containing both self-administered questionnaires were posted on-line via several healthcare workers’ WhatsApp platforms of various health facilities. The two questionnaires were formatted in such a way that all questions were required to be answered for submission to be possible. The completely filled questionnaires were submitted via Google to the attached e-mail address.

### Data analysis

All the data received via Google within the specified period of data collection were cleaned and analysed with SPSS version 23 software. Data were summarised with descriptive statistics such as mean, standard deviation and percentage. The chi-square test was used to investigate associations between psychological distress and socio-demographic, level of knowledge of COVID-19 and work-related variables. Variables that were significant in the chi-square analysis were entered into a binary logistic regression model to further explore the association between psychological distress and level of knowledge whilst controlling for the effect of other confounding variables such as gender and contact with COVID-19 positive patients. All analyses were carried out at 5% level of significance.

### Ethical considerations

Informed consent and confidentiality were ensured. Ethical approval was given by Ethics and Research Committee, Ladoke Akintola University of Technology Teaching Hospital Osogbo, Osun State, protocol number: LTH/EC/2020/07/465.

## Results

A total of 313 completed questionnaires were received and analysed. Of the 313 respondents, 147 (47%) had a GHQ-12 score of 3 and above indicating psychological distress, whilst 166 (53.0%) scored less than 3.

### Association between psychological distress and socio-demographic variables

Being female was the only socio-demographic variable (χ^2^ = 7.07, *p* = 0.008) that was found to be significantly associated with psychological distress (Table 1).

**TABLE 1:** Association between psychological distress and socio-demographic variables.

Variable	Psychological distress						χ^2^	*p*
	Total (*N* = 313)		GHQ-12 < 3 (*n* = 166)		GHQ-12 ≥ 3 (*n* = 147)			
	*n*	%	*n*	%	*n*	%		
**Age**							1.71	0.653
≤ 30	85	27.2	48	56.5	37	43.5	-	-
31–40	107	34.2	57	53.3	50	46.7	-	-
41–50	103	32.9	50	48.5	53	51.5	-	-
> 50	18	5.8	11	61.1	7	38.9	-	-
**Gender**							7.07	0.008
Male	157	50.2	95	60.5	62	39.5	-	-
Female	156	49.8	71	45.5	85	54.5	-	-
**Religion**							1.91	0.168
Christian	162	51.2	92	56.8	70	47.6	-	-
Islam	151	48.2	74	49.0	77	51.0	-	-
**Marital status**							0.01	0.911
Not married	80	25.6	42	52.5	38	47.5	-	-
Married	233	74.4	124	53.2	109	46.8	-	-
**Frequency of participation in religious activities**							0.85	0.358
Frequent	276	88.2	149	54.0	127	46.0	-	-
Not frequent	37	11.8	17	45.9	20	54.1	-	-

GHQ, General Health Questionnaire.

### Association between psychological distress and work-related variables

Of the work-related variables, only having contact with COVID-19 positive patients was significantly associated with psychological distress (χ^2^ = 5.57, *p* = 0.018) (Table 2).

**TABLE 2:** Association between psychological distress and work-related variables.

Variables	Psychological distress						χ^2^	*p*
	Total (*N* = 313)		GHQ-12 < 3 (*n* = 166)		GHQ-12 ≥ 3 (*n* = 147)			
	*n*	%	*n*	%	*n*	%		
**Type of healthcare worker**							4.45	0.486
Nurses	28	8.9	11	39.3	17	60.7	-	-
Doctors	166	53.0	92	55.4	74	44.6	-	-
Medical laboratory scientists	68	21.7	38	55.9	30	44.1	-	-
Pharmacists	12	3.8	6	50.0	6	50.0	-	-
Radiographers	7	2.2	2	28.6	5	71.4	-	-
Others	32	10.2	17	53.1	15	46.9	-	-
**Years of service (years)**							2.92	0.571
< 5	83	26.5	42	50.6	41	49.9	-	-
5–10	59	18.8	34	57.6	25	42.4	-	-
11–15	93	29.7	52	55.9	41	44.1	-	-
16–20	42	13.4	23	54.8	19	45.2	-	-
> 20	36	11.5	15	41.7	21	58.3	-	-
**Contact with COVID**-**19 positive patient**							5.57	0.018
Yes	176	56.2	83	47.2	93	52.8	-	-
No	137	43.8	83	60.6	54	39.4	-	-
**Recovery or death of patient**							3.85	0.146
Recovered	111	35.5	51	45.9	60	54.1	-	-
Died	18	5.8	9	50.0	9	50.0	-	-
I don’t know	184	58.8	106	57.6	78	42.4	-	-
**Being a routine frontline worker**							2.09	0.148
Yes	123	39.3	59	48.0	64	52.0	-	-
No	190	60.7	107	56.3	83	43.7	-	-
**Provision of incentives as a frontline worker**							0.06	0.803
Yes	16	5.1	8	50.0	8	50.0	-	-
No	297	94.9	158	53.2	139	46.8	-	-

COVID-19, coronavirus disease 2019.

GHQ, General Health Questionnaire.

### Association between psychological distress and knowledge

Psychological distress was more frequent amongst healthcare workers with a good knowledge of COVID-19 (*n* = 52; 57.1%) than those with a poor level of knowledge (95; 42.8%), χ^2^= 5.34, *p* = 0.021 (Table 3).

**TABLE 3:** Association between psychological distress and knowledge of healthcare workers about coronavirus 2019.

Variables	Psychological distress						χ^2^	*p*
	Total (*N* = 313)		GHQ-12 < 3 (*n* = 166)		GHQ-12 ≥ 3 (*n* = 147)			
	*n*	%	*n*	%	*n*	%		
**Knowledge**								
Good	91	29.1	39	42.9	52	57.1	5.34	0.021
Poor	222	70.9	127	57.2	95	42.8	-	-

GHQ, General Health Questionnaire.

### Logistic regression analysis for psychological distress

Female healthcare workers were about twice as likely to have psychological distress as their male counterparts (OR = 1.92, 95% CI: 1.21–3.04, *p* = 0.006). Healthcare workers who had no contact with COVID-19 positive patients had an 87% reduced risk of psychological distress (OR = 0.13, 95% CI: 0.23–0.25, *p* = 0.018) compared with those who had contact with COVID-19 positive patients. Healthcare workers with poor knowledge of COVID-19 had a 44% reduced risk of psychological distress (OR = 0.56, 95% CI: 0.34–0.93, *p* = 0.025) compared with those with good knowledge (Table 4).

**TABLE 4:** Logistic regression for psychological distress and selected variables.

Variables	OR	*p*	95%CI	
			Lower	Upper
**Gender**				
Male (reference)	-	-	-	-
Female	1.92	0.006	1.21	3.04
**Contact with COVID-19 positive patient**				
Yes (reference)	-	-	-	-
No	0.13	0.018	0.23	0.25
**Knowledge**				
Good (reference)	-	-	-	-
Poor	0.56	0.025	0.34	0.93

COVID-19, coronavirus disease 2019; OR, odds ratio; CI, confidence interval.

## Discussion

The prevalence of psychological distress amongst respondent healthcare workers was 47.0%. Female gender was a risk factor whilst poor knowledge of the pandemic and lack of contact with of COVID-19 positive patients were protective factors.

### Prevalence of psychological distress amongst healthcare workers in Nigeria

Nearly half of the participants reported psychological distress in this study. This is comparable with a study in Pakistan where a prevalence of 42% for moderate psychological distress was reported amongst healthcare workers.^17^

However, in China, 71.5% of healthcare workers reported distress compared with the 47% in our study. The lower prevalence in our study may be related to differences in the study population, as only healthcare workers attending to patients with fever or COVID-19 clinic participated in the Chinese study. The difference in the instruments used may also have a bearing on the results. Psychological distress in our study was measured with GHQ-12 whilst distress in the Chinese study was assessed with the 22-item Impact of Event Scale–Revised. Although both are self-report questionnaires, the 22-item Impact of Event Scale–Revised is more specific to traumatic events whilst GHQ-12 measures general psychiatric morbidity. Another reason for the lower prevalence in our study may be because of previous knowledge of the pandemic before it found its way into the country. Coronavirus disease 2019 was reported in China at the end of 2019, whilst the first case ever reported in Nigeria was on 27 February 2020.^1,2,18^

Religion has been shown to influence behaviour through the control of emotional response to stressors or through the modification of the social milieu of the stressor itself.^19^ Given the high number of respondents who participated in religious activities in our study, religion may be a good coping mechanism ameliorating the effect of the pandemic on healthcare workers. However, there was no significant difference between those who participated frequently in religious activities and those who did not.

Prior to COVID-19 pandemic in Nigeria, studies carried out amongst healthcare workers with GHQ-12 in 2012 and 2018 reported prevalence of 10% and 17.2%, respectively. Therefore, the higher prevalence in our study may not be unconnected with COVID-19 pandemic. The 2012 study was conducted amongst 30 doctors and nurses working at a university medical centre whilst the 2018 survey was carried out amongst 256 doctors, nurses, pharmacists and medical laboratory scientists working in a teaching hospital.^20,15^

### Psychological distress and socio-demographic variables

Psychological distress was more in females compared with males. In keeping with our study, women reported more severe symptoms in China.^9,18,21^ Similarly, in Saudi Arabia, the level of distress was higher amongst females.^22^ Women suffer more from psychological distress than men, especially when confronted with clashes between their jobs and family lives.^23,24^ The novelty of the pandemic might have disrupted the hitherto equilibrium between work and family life achieved amongst females.

Another explanation could be that men may not be too willing to admit to some of the symptoms elicited by GHQ-12 that are not in keeping with their conventional role in the society.^25^ Given the non-availability of data base of all healthcare workers, the possibility of having a higher ratio of women to men amongst all healthcare workers in Nigeria amongst those who participated in the study cannot be verified. Otherwise, the probability of a higher ratio of women compared with men would have been said to be the reason why more women were distressed. However, it is good to note that, the probability of having a higher proportion of women with psychological distress out of those who actually participated in our study could not have been related to their higher number given that the number of male and female respondents in this study were close.

### Psychological distress and work-related variables in Nigeria

No significant association was found between psychological distress and different categories of healthcare workers, including nurses. This is contrary to other literature findings where nurses were reported to be at increased risk of mental health challenges during outbreak of infectious diseases.^26^ Studies have shown that the female gender and being nurses who invariably are females are risk factors for psychological distress.^9,18,27^ It is, however, noteworthy that in our study, female gender is a risk factor, whilst being a nurse is not.

It is not surprising that psychological distress was more amongst healthcare workers who had contact with COVID-19 positive patients. Coming into proximity with people infected by the virus may increase the fear of contracting the disease, which may lead to distress. This risk may be compounded by pre-existing poor healthcare system, poor funding and scarcity of personal protective equipment, which has been a cause for concern in some healthcare delivery systems.^18,28,29^

### Psychological distress and knowledge of coronavirus 2019

The number of participants with good knowledge of COVID-19 who developed psychological distress was higher compared with those with poor knowledge. This is a paradoxical finding. Good knowledge is assumed to be protective, however, those with good knowledge were the ones more affected. Alternative explanation may be that knowledge about the cause and prevention of COVID-19 may stimulate negative emotional responses more, especially when it is seemingly impossible to achieve the preventive measures in a poor resource setting.

In addition, El Hage et al. in a narrative review of literature on mental health risks of COVID-19 amongst healthcare workers identified lack of specific drugs as contributory to the mental health problems amongst healthcare workers.^30^ Furthermore, the absence of a vaccine for the containment of the virus reported by Cascella et al. in a review of the features, evaluation and treatment of COVID-19 maybe a source of distress.^31^ It may therefore not be out of place when healthcare workers who have a good knowledge of COVID-19 are more distressed.

## Limitations and strength

Causality cannot be determined because of the cross-sectional nature of the study. Data were a convenient sample, which may impact its representativeness. In addition, use of self-report questionnaires may give room to report bias from respondents. There is, therefore, a need to exercise caution in the interpretation of our findings in the light of these limitations. This is the first on-line study on psychological distress amongst healthcare workers in Nigeria during COVID-19 pandemic to the best of our understanding.

## Conclusion

Our findings revealed that healthcare workers in Nigeria reported psychological distress during COVID-19, with greater risk amongst females compared with males and amongst those who had contact with COVID-19 positive patients. Unexpectedly, poor knowledge was found to be protective. There is a need for more research to evaluate the role of the pandemic in the reported distress, as well as risk and protective factors.
